# The effect of direct access to CT scan in early lung cancer detection: an unblinded, cluster-randomised trial

**DOI:** 10.1186/s12885-015-1941-2

**Published:** 2015-11-25

**Authors:** Louise Mahncke Guldbrandt, Morten Fenger-Grøn, Torben Riis Rasmussen, Finn Rasmussen, Peter Meldgaard, Peter Vedsted

**Affiliations:** Research Centre for Cancer Diagnosis in Primary Care, Research Unit for General Medical Practice, Aarhus University, Bartholins Alle 2, 8000 Aarhus, Denmark; Section for General Medical Practice, Department of Public Health, Aarhus University, Aarhus, Denmark; Department of Pulmonary Medicine, Aarhus University Hospital, Aarhus, Denmark; Department of Radiology, Aarhus University Hospital, Aarhus, Denmark; Department of Oncology, Aarhus University Hospital, Aarhus, Denmark

## Abstract

**Background:**

Lower lung cancer survival rates in Britain and Denmark compared with surrounding countries may, in part, be due to late diagnosis. The aim of this study was to evaluate the effect of direct access to low-dose computed tomography (LDCT) from general practice in early lung cancer detection on time to diagnosis and stage at diagnosis.

**Methods:**

We conducted a cluster-randomised, controlled trial including all incident lung cancer patients (in 19-month period) listed with general practice in the municipality of Aarhus (300,000 citizens), Denmark. Randomisation and intervention were applied at general practice level. A total of 266 GPs from 119 general practices. In the study period, 331 lung cancer patients were included. The intervention included direct access to low-dose CT from primary care combined with a 1 h lung cancer update meeting. Indication for LDCT was symptoms or signs that raised the GP’s suspicion of lung cancer, but fell short of satisfying the fast-track referral criteria on red flag’ symptoms.

**Results:**

The intervention did not significantly influence stage at diagnosis and had limited impact on time to diagnosis. However, when correcting for non-compliance, we found that the patients were at higher risk of experiencing a long diagnostic interval if their GPs were in the control group.

**Conclusion:**

Direct low-dose CT from primary care did not statistically significantly decrease time to diagnosis or change stage at diagnosis in lung cancer patients. Case finding with direct access to LDCT may be an alternative to lung cancer screening. Furthermore, a recommendation of low-dose CT screening should consider offering symptomatic, unscreened patients an access to CT directly from primary care.

**Trial registration:**

www.clinicaltrials.gov, registration ID number NCT01527214.

## Background

Lung cancer is the most common cause of cancer death in the industrialised world [1]. The stage of the disease profoundly predicts survival. Efforts to diagnose lung cancer early have included screening initiatives [2, 3] and expedited investigation of symptoms indicative of lung cancer [4–6]. Although screening might reduce mortality from lung cancer in relative terms, it remains very doubtful that such a strategy will be implemented worldwide.

So far, most lung cancer patients with symptomatic disease present to the general practitioner (GP) before diagnosis [7, 8]. Symptoms that may indicate lung cancer are common in primary care [9]. GPs must distinguish between those few patients whose symptoms are due to lung cancer and the large group of patients who have benign disease [10]. For many of these symptomatic patients, the ideal strategy might not be a full fast-track referral, but easy access to a relevant investigation in general practice.

Patients in Britain and Denmark have lower survival from lung cancer and fewer are diagnosed in the early stages compared with other European countries (11). Much effort has therefore been devoted to achieving earlier diagnosis of lung cancer. British and Danish initiatives to expedite cancer diagnosis have included clinical initiatives (e.g. National Institute for Health and Care Excellence (NICE) guidelines for symptoms in the UK) as well as organisational initiatives (e.g. fast-track referral pathway in Denmark [11, 12]).

However, essentially three difficulties have been revealed; a significant proportion of lung cancer patients present with unspecific symptoms with low positive predictive values [13], the majority of lung cancer patients are diagnosed by other routes than the expedited one [14, 15], and there may be a need for a technological update of the primary diagnostic investigation which has been the chest X-ray. Thus, 15–23 % of all new lung cancer patients have had a false-negative chest X-ray before diagnosis, which has important implications for the time to diagnosis [16] and challenges the traditional primary care investigation. A low-dose computed tomography (LDCT), on the other hand, has proven to have a high sensitivity for lung cancer [17], even in early-stage cancer. However, we do not know whether direct access to LDCT will optimise lung cancer diagnosis in symptomatic patients seen by their GPs.

We hypothesised that GPs who had direct access to LDCT and received an update on lung cancer detection would diagnose lung cancer faster and at earlier stages. Furthermore, we hypothesised that this intervention would increase the GPs’ awareness of lung cancer and make them increase their standard investigations (e.g. fast-track use) for lung cancer.

The aim of this study was to evaluate the effect of direct access to fast low-dose chest CT combined with specific training in the diagnosis of lung cancer in general practice on the time to diagnosis and the stage at diagnosis. Furthermore, we wanted to evaluate differences between the intervention practices’ and the control practices’ use of fast-track investigation.

## Methods

We conducted a cluster-randomised, controlled, two-arm (1:1), unblinded study. The intervention was a technological upgrade comprising direct access to chest low-dose CT combined with a simple continuing medical education (CME) meeting on lung cancer diagnostics in general practice. The study took place in Aarhus municipality, Denmark, during a 19-month period from November 2011 to June 2013.

Denmark has a tax-financed healthcare system with free access to medical advice and treatment. Approximately 99 % of Danish citizens are registered with a GP whom they must consult for medical advice. GPs act as gatekeepers to investigations and hospitals with a few exceptions, e.g. emergencies.

Before November 2011, the GPs in the area had three diagnostic work-up possibilities for patients with respiratory symptoms that could indicate lung cancer. They could either refer patients to 1) an X-ray, 2) the Department of Pulmonary Medicine within the normal waiting list, or 3) the lung cancer fast-track pathway with a maximum of 72 h’ waiting time. Indication for fast-track was either an abnormal chest X-ray or certain qualifying ‘red- flag’ symptoms (e.g. coughing (>four weeks) or haemoptysis) [18]. GPs were not allowed to refer directly to a CT.

### Participants

**Fig. 1:**
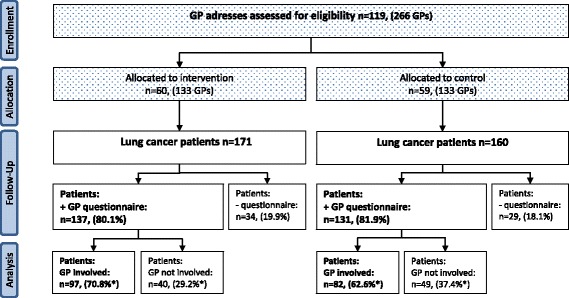
Participants flow. *Percentage of patients with questionnaire data

A total of 119 general practices in the catchment area of a single department of pulmonary medicine, Aarhus University Hospital, with 266 GPs were randomised into two groups (Fig. 1). At patient level, the inclusion criteria were that the patient was on the list of a participating GP during the study period and had a recent diagnosis of lung cancer (ICD10 34.0–9). There were no exclusion criteria.

### Randomisation

The randomisation was performed by a data manager who used Stata 12.0. The 119 practices were allocated a random number between zero and one and then listed from the lowest to the highest value. The top 60 practice addresses (133 GPs) formed the intervention group with practice addresses as cluster level.

### Intervention

The intervention was allocated at the cluster level. Six times within an initial 3-month period, the intervention practices were informed by letter about the intervention. The letters included information concerning the referral procedures and indications for the CTs. The indication was the GP’s suspicion that the patient’s signs and symptoms could possibly be related to lung cancer. Excepted from this were patients who met the indication for fast-track pathway referral who should be referred to the fast-track as usual.

The GPs were offered participation in a 1 h small-group-based CME meeting held during the first 2 months of the study to increase their awareness of early lung cancer and to encourage them to refer more patients to tests (LDCT scan or fast-track pathway) for lung cancer. During the meeting, the GPs were briefed about the state-of-the-art on early detection of lung cancer based on algorithms for positive predictive values in primary care [13, 19]. The GPs also received information about the use of CT and how to interpret CT reports. The education was developed and conducted by PV and LMG.

Controls were not informed about the study and they hence continued referring their patients as usual.

The Department of Radiology, Aarhus University Hospital, carried out the CTs. The scans were performed on a Brilliance 64, Philips, Best, the Netherlands: collimation 64 × 0.625, slice thickness 2 mm, increment 1 mm, pitch 1, rotation time 0.75 sec. The effective radiation dose (Monte Carlo Simulation Software, CT-Expo v. 2.1) was 2–3 mSv. Intravenous contrast medium was not administered. The waiting time limit from referral to performed CT was a maximum of two working days.

The CT reports were made by three sub-specialised radiologists. Based on the CT report and the patient’s medical history, a recommendation was compiled at a conference between a chest physician and a radiologist the day after the scan, and forwarded electronically to the GP. The GP had full responsibility for informing the patient about the result and, if necessary, to refer the patient for further diagnostic work-up.

If lung nodules (4–10 mm) that could not be categorised as benign were detected, the GP was informed to refer the patient to a follow-up program (3, 6 or 12 months after the first scan) according to the size and the characteristics of the nodules following the international standard [20]. Incidental findings on the CT scan outside the lungs judged to be of clinical significance were reported to the GP with recommendations for referral to a relevant department.

If the CT scan revealed any suspicion of lung cancer, the patients were referred by the GP through the fast-track pathway to standard diagnostic work-up at the Department of Pulmonary Medicine. The diagnostic work-up included contrast enhanced CT (including PET if surgery was an option). Furthermore, a diagnosis was obtained by either bronchoscopy with biopsy, fine-needle aspiration (FNA) in association with endoscopic ultrasound or endobronchial ultrasound, or transthoracic FNA. The final clinical staging of lung cancer was provided by a multi-disciplinary team decision according to the 7th TNM Classification of Malignant Tumors [21]. Early-stage patients (stage I-IIb) were offered surgical resection according to Danish guidelines.

### Sample size

It can be assumed that lung cancer patients are randomly distributed among GPs. However, the incidence of lung cancer could be higher in some areas with many smokers and in practices with many elderly patients. To account for an unknown intra-cluster correlation coefficient (ICC), we calculated a design effect of 1.25 [22].

In 2008 half of the Danish lung cancer patients waited 33 days or more (the median) from first presentation to primary care to diagnosis of lung cancer [23]. We wanted to be able to show a decrease in the diagnostic interval to a level where only 25 % of the patients have to wait 33 days or more. With a one-sided alpha of 5 % and a power of 80 %, we had to include 54 lung cancer patients in each arm with a 1:1 randomisation. Given the design effect, we had to include a total of 135 lung cancer patients with questionnaire data and GP involvement in the diagnosis.

### Harms

This intervention offered the GP an update on lung cancer and direct access to a CT scan. If the GP or the patient did not want to participate, they could always choose not to. A potential harm was the extent of nodules and incidental findings in the scans that may lead to further examinations which ultimately could turn out to be unnecessary if the findings were benign. The number of derivative investigations after the low-dose CT scan has been published previously [24].

### Outcomes

Primary outcomes were the primary care interval and the diagnostic interval. The primary care interval is defined as the time from the patient’s first presentation in primary care until referral to secondary care; the diagnostic interval is defined as the time from the first presentation until decisive diagnosis [25].

The secondary outcome was the stage at diagnosis as stated in a multidisciplinary team’s decision on the clinical TNM (cTNM) stage. The cancer stage was re-grouped into stage IA, 1B, IIA, IIB, IIIA, IIIB and IV from the TNM (version 7). The stage was then dichotomised into local and advanced using a cut-point between stage IIIA and IIIB. This was done as there is a significant difference in mortality between these two stages [26].

As a naturally derived effect of the new diagnostic modality combined with a CME focusing on lung cancer diagnosis, we wanted to test whether there was a difference in the use of the existing fast-track and the PPV for lung cancer in the fast-track between intervention and control GPs. Patients referred to fast-track evaluation for lung cancer were coded DZ 03.1B (lung cancer observation). This code, combined with a unique GP number, gave information about referral to the fast-track pathway.

### Variables and data sources

All cases of lung cancer (ICD10 34.0–9) were identified starting from 1 January 2012. To ensure completeness, cases were obtained from a combined identification in the Danish Lung Cancer Registry (DLCR) and the Danish National Patient Registry (NPR). The lung cancer cases were checked against practice patient lists in order to identify the patients’ GPs. From these lists, we also gathered information about the size of the practice lists and the age and gender distributions of the patients listed with each practice.

The DLCR was established in 2001 as a national database. Since 2003, data have covered more than 90 % of all lung cancer cases in Denmark [27]. Registrations are made electronically and no later than two months after diagnosis. The NPR is a national population-based database containing admission/discharge dates and discharge diagnosis (classified according to the International Classification of Diseases, ICD-10) on all inpatient, outpatient clinics and emergency room visits at Danish hospitals [28].

We used the Danish Deprivation Index (DADI) to gather information about deprivation level in the different GP clinics’ populations. The index consists of eight variables registered in Statistics Denmark for all citizens [29]. The DADI data are expressed numerically as a value between 10 and 100; the higher the number, the more deprived the practice population. The variables used are: (i) Proportion of adults aged 20–59 with no employment, (ii) proportion of adults aged 25–59 with no professional education, (iii) proportion of adults aged 25–59 with low income, (iv) proportion of adults aged 18–59 receiving public welfare payments (transfer income or social benefits), (v) proportion of children from parents with no education and no professional skills, (vi) proportion of immigrants, (vii) proportion of adults aged 30+ living alone and (viii) proportion of adults aged 70+ with low income (= the lowest national quartile).

Data on patient comorbidity were obtained from a GP questionnaire in which the GP stated if comorbidity was present or not. Data on each identified lung cancer patient’s socio-economic position were collected from Statistics Denmark. Education included basic school and was dichotomised into “≤10 years” and “>10 years” [30]. Marital status was dichotomised into “cohabitating” or “living alone”.

The Danish civil registration number (CRN), a unique 10-digit personal identification number, was used to link registers [31].

### GP questionnaire

A questionnaire was sent to the lung cancer patient’s general practice. In practices with more than one GP, we asked the GP most familiar with the patient to complete the questionnaire. The GPs were told to use their medical records when answering questions about whether the general practice/GP had been involved in the diagnosis of the lung cancer, together with the dates in the diagnostic pathway and the use of a fast-track pathway. The questionnaire was based on previously used and validated items [16, 23, 25, 32]. Non-responders received a reminder after 4 weeks. The responding doctors got a reimbursement for participation (€17, £15).

### Statistical methods

Pearson’s chi-squared test or Wilcoxon rank-test were used for comparison of patients listed with either intervention or control GPs in terms of baseline characteristics as well as for the crude analysis of study outcomes.

Primary analyses were by standard intention-to-treat with participants analysed according to their GP’s randomisation. The primary care and the diagnostic interval are presented as medians with inter-quartile intervals (IQI). We used general linear models (GLM) for the binomial family to calculate associations between long intervals and the patients’ randomisation status. Long intervals were defined as the 4th quartile of similar intervals from Danish lung cancer patients in 2010 [33]. In these analyses, we accounted for clusters of patients within GPs using cluster robust variance estimation and adjusted for patient age and presence of comorbidity as it has previously been shown that these factors can influence the length of the intervals [33].

In supplementary analyses, we corrected for non-compliance by comparing patients from GPs who participated in the CME with patients from a similar group of patients from control GPs [34]. These estimates are not diluted by lack of compliance as they are standard in intent-to-treat analyses.

Referral rates were calculated based on the number of patients referred by the GP per project month per patient aged 25 years and above. For the non-compliance analyses on referral rates, we used the risk of having a low referral rate (defined as among the 25 % lowest referral rates for the two groups together).

Estimates were presented with 95 % confidence intervals (95 % CI) when relevant. Analyses were made using Stata 12.0.

### Numbers analysed

Descriptive analyses and analyses on cancer stage were performed for the entire study population. The analysis of time intervals was restricted to patients for whom the GP returned the questionnaire and for whom the GP was involved in the diagnosis.

### Ethics

The study was approved by the Danish Data Protection Agency (ref. no.: 2011-41-6872) and the Danish Health and Medicines Authority (ref. no.: 7-604-04-2/357/KWH). According to the Research Ethics Committee of the Central Denmark Region, the Danish Act on Research Ethics Review of Health Research Projects did not apply to this project (ref.no.: 118/2011) as CT was already a widely used technology. The study is registered at Clinical Trials (www.clinicaltrials.gov: NCT01527214).

## Results

### Participants flow

During the study period, 331 incident lung cancer patients were diagnosed; 171 were listed with intervention GPs and 160 with control GPs (Fig. 1). In the intervention group, 80.1 % (137 patients) of the GP questionnaires were returned; in the control group, 81.9 % (131 patients). Intervention GPs were involved in the lung cancer diagnosis of 97 patients (70.8 %, 95 % CI: 62.4–78.3), whereas the GPs in the control group were involved in the diagnosis of 82 patients (62.6 %, 95 % CI: 53.7–70.9) (*p* = 0.154) (Fig. 1).

### Baseline data

The GPs in the intervention group were slightly older (mean 53.6 years compared with 51.6 years), their patients were slightly more deprived and more were working in a solo practice (Table 1). Sixty-four (48.5 %) of the GPs who were offered CME participated.

**Table 1:** Baseline characteristics of GPs in the control and intervention groups. Numbers with (percentages) unless stated otherwise

	GP intervention		GP control	
All GPs	133		133	
All practice	60		59	
Gender:				
Male	68	(51.1)	64	(48.1)
Female	65	(48.9)	69	(51.9)
Age:				
Mean (min-max)	53.6	(38–68)	51.6	(38–65)
38–45 yrs.	26	(19.5)	23	(17.3)
46–55 yrs.	67	(50.4)	52	(39.1)
56–68 yrs.	40	(30.1)	58	(43.6)
Practice type:				
Solo	21	(35.0)	16	(27.1)
2 or more	39	(65.0)	43	(72.9)
List size/GP^a^, Median (range)	1008	(585–2780)	992	(500–3347)
DADI^b^, Median (IQR)	25.4	(20.5–31.6)	22.5	(18.5–30.8)
CME participation	64	(48.5)		

^a^List size per GP (patients aged ≥25 years). ^b^Danish Deprivation Index (min:10-max:100)

Lung cancer patients from the intervention and the control GPs were similar with respect to age, education, marital status and comorbidity, while the control group had a higher proportion of women (Table 2). No statistically significant differences between the intervention and the control group patients for which the GPs returned the questionnaire were observed (Appendix).

**Table 2:** Characteristics of the lung cancer patients. All patients, patients from control and intervention GPs, and patients for whom the GP was involved in the diagnosis of the lung cancer

	All patients		Intervention all		Control all		*P*-value	Intervention, GP involved		Control, GP involved		*p*-value
	n	(%)	n	(%)	n	(%)		n	(%)	n	(%)	
All: n	331		171	(51.7)	160	(48.3)		97	(70.8)	82		
Gender:												
Male	157	(47.4)	91	(53.2)	66	(41.3)	0.029^a^	46	(47.4)	40	(48.8)	0.459^a^
Female	174	(52.6)	80	(46.8)	94	(58.7)		51	(52.6)	42	(51.2)	
Age:												
Mean (95%CI)	69.4	(68.4–70.5)	69.6	(68.1–70.5)	69.3	(67.7–70.8)	0.799^a^	69.8	(67.8–71.7)	68.3	(66.2–70.4)	0.830^b^
Range		40–97 yrs.		40–97 yrs.		44–86 yrs.			45–89 yrs.		44–86 yrs.	
40–59 yrs	61	(18.4)	33	(19.3)	28	(17.5)	0.904^b^	19	(47.5)	16	(19.5)	0.440^c^
60–79 yrs	222	(67.0)	111	(64.9)	111	(69.4)		63	(64.9)	59	(72.0)	
80+	48	(14.5)	27	(15.8)	21	(13.1)		15	(15.4)	7	(8.5)	
Education:												
<10 yrs	136	(41.1)	67	(39.2)	69	(43.1)	0.733^b^	35	(36.1)	37	(45.1)	0.434^c^
10–15	146	(44.1)	77	(45.0)	69	(43.1)		45	(46.4)	33	(40.3)	
>15 yrs	40	(12.1)	20	(11.7)	20	(12.5)		13	(13.4)	11	(13.4)	
Unknown	9	(2.7)	7	(4.1)	2	(1.3)		4	(4.1)	1	(1.2)	
Marital status												
Cohabitating	172	(52.0)	88	(51.5)	84	(52.5)	0.850^a^	55	(56.7)	44	(53.7)	0.422^a^
Living alone	159	(48.0)	83	(48.5)	76	(47.5)		42	(43.3)	38	(46.3)	
Comorbidity -questionnaire												
Yes	231	(69.8)						83	(85.6)	69	(84.1)	0.663^a^
No	35	(10.6)						13	(13.4)	13	(15.9)	
Unknown	65	(19.6)						1	(1.0)	0		

^a^Differences between groups were tested by Pearsons χ^2^ test. ^b^Age difference between groups were tested by Student’s *T*-test. ^c^Diffrences between groups were tested by Wilcoxon test

### Primary outcomes: the primary care and the diagnostic intervals

For all patients, the median primary care interval was 16 days (IQI: 4–56) (Table 3). There was no statistically significant difference in primary care interval between patients in the intervention group (median: 14 days, IQI: 4–53) and patients in the control group (median: 18 days, IQI: 5–69).

**Table 3:** Primary care and diagnostic intervals (in days) for lung cancer patients with a referral route involving the GP, according to groups. Only GP-involved patients are included in the analyses. Adjusted and unadjusted associations for long intervals (the 75 percentile from 2010) are presented as prevalence ratios (PRs) with 95 % confidence intervals (95 % CI)

	Primary care interval:							Diagnostic interval:						
	N	Median	IQI	*p*-value	Prevalence of long interval	PR (95 % CI) Unadjusted	PR (95 % CI) adjusted^a^	N	Median	IQI	*p*-value	Prevalence of long interval	PR (95 % CI) Unadjusted	PR (95 % CI) adjusted^a^
All	155	16	4–56		36.1 (28.6–44.2)			160	39	17–93		33.1 (25.9–41.0)		
Controls	74	18	5–69		36.5 (25.6–48.5)	1	1	75	44	17–112		37.3 (26.4–49.3)	1	1
Intervention	81	14	4–53	0.455^d^	35.8 (25.4–47.2)	0.98 (0.65–1.49)	0.99 (0.65–1.54)	85	36	17–83	0.299^d^	29.4 (20.0–40.3)	0.79 (0.51–1.23)	0.80 (0.50–1.27)
Intervention:														
- CME^b^	32	37	8–61		53.1 (34.7–70.9)	1	1	35	66	22–143		42.9 (26.3–60.6)	1	1
+ CME^c^	49	9	3–27	0.048^d^	24.5 (13.3–38.9)	0.46 (0.26–0.83)	0.49 (0.27–0.88)	50	23	15–50	0.008^d^	20.0 (10.0–33.7)	0.47 (0.24–0.92)	0.45 (0.20–1.03)

^a^Adjusted for patient age and co-morbidity (yes/no). Clusters are accounted for. ^b^The GPs who did not participate in CME or who did not work in a clinic with a GP who participated. ^c^The GPs participating in CME or working in a clinic with a participating GP. ^d^Differences between groups were tested by Wilcoxon test

The overall median diagnostic interval was 39 days (IQI: 17–93). Patients listed with control GPs had a statistically insignificantly longer median diagnostic interval (44 days, IQI: 17–112) than patients listed with intervention GPs (36 days, IQI: 17–83) (*p* = 0.299).

There was no difference in the proportions experiencing long primary care or diagnostic intervals between patients from the control and the intervention groups. Within the intervention group, both primary care and diagnostic intervals were statistically significantly shorter if the GP (or a GP in the clinic) participated in the CME (primary care interval median: 9 days vs. 37 days, *p* = 0.048; diagnostic interval median: 23 vs. 66, *p* = 0.008).

Correcting for non-compliance, we found a statistically insignificantly higher risk for having a long diagnostic interval for patients from the control group (risk difference (RD): 13.5 % (95 % CI:−11.0–37.9 %, *p*-value = 0.280)). No difference in risk for having a long primary care interval was observed using this approach (RD: 1.1 % (95 % CI: 123.9–26.1 %, *p*-value = 0.929)).

### Secondary outcomes: stage

A total of 41.4 % of all lung cancer patients were in a localised stage. There was no difference in stage distribution between patients from the control and the intervention GPs in the non-adjusted analyses (Table 4). We found no difference in the risk of having localised stage when adjusting for non-compliance (RD: 1.5, 95 % CI:−31.8–34.9, *p* value = 0.927).

**Table 4:** Stage distribution for patients in the study (all patients dived between intervention and controls), and only for patients for whom the GP was involved in the diagnosis

	All patients		Controls, all		Intervention, all		*P*-value	Controls, involved		Intervention, involved		*p*-value
	n	(%)	n	(%)	n	(%)		n	(%)	n	(%)	
Stage IA	46	(13.9)	19	(11.9)	27	(15.8)		4	(4.9)	11	(11.3)	
Stage IB	33	(10.0)	18	(11.3)	15	(8.8)		8	(9.8)	8	(8.3)	
Stage IIA	14	(4.2)	3	(1.9)	3	(1.8)		3	(3.7)	3	(3.1)	
Stage IIB	22	(6.6)	11	(6.9)	11	(6.4)		7	(8.5)	7	(7.2)	
Stage IIIA	22	(6.6)	9	(5.5)	13	(7.6)		5	(6.1)	6	(6.2)	
Stage IIIB	24	(7.3)	12	(7.5)	12	(7.0)		7	(8.5)	9	(9.3)	
Stage IV	170	(51.4)	88	(55.0)	90	(52.6)	0.586^a^	48	(58.5)	53	(54.6)	0.470^a^
Localised (IA-IIIA)	137	(41.4)	60	(37.5)	69	(40.4)	0.595^b^	27	(32.9)	35	(36.1)	0.658^b^
Metastatic (IIIB-IV)	194	(58.6)	100	(62.5)	102	(59.6)		55	(67.1)	62	(63.9)	

^a^Differences between groups were tested by Wilcoxon test.^b^Differences between groups were tested by Pearsons χ^2^ test

### General effects on other diagnostic stategies

The GPs referred 836 patients to the lung cancer fast-track during the study period which resulted in 81 lung cancer diagnoses. This corresponds to a PPV of 9.7 % (10.1 % for control GPs and 9.4 % for intervention GPs; *p*-value: 0.732) for lung cancer diagnosed via the fast-track lung cancer pathway. The unadjusted referral rate to fast-track was 0.17 per 1000 adults listed per GP per month (95 % CI: 0.12–0.25) for intervention patients compared with 0.15 (95 % CI: 0.11–0.24) for control GPs (*p*-value: 0.417). When correcting for non-compliance, we found no difference in PPV between the two groups (RD: 1.1 % (95 % CI:−5.8–8.2, *p* = value: 0.740)), but a statistically insignificantly higher risk for having a low referral rate (below the lowest referral rate quartile) to fast-track for control GPs (RD: 6.3 % (95 % CI:−22.7–35.3, *p*-value: 0.670)).

## Discussion

### Main results

In a cluster-randomised trial with a combination of CME and direct access to LDCT, we found no statistically significant difference in primary care or diagnostic intervals between patients listed with the control and the intervention GPs. However, when correcting for non-compliance, we found that the patients were at higher risk of experiencing a long diagnostic interval if their GPs were in the control group. There was no difference between the groups in terms of stage, the use of the fast-track pathway or the PPV for lung cancer.

### Strength and limitations

This study is, to the authors’ knowledge, the first randomised controlled trial testing the effect of direct access to low-dose CT from primary care. The study design of randomising by clusters at the level of general practice address (and not at the level of patient or clinician) was appropriate to the research question. It would not be possible to ask the GP to allocate individual patients randomly, and allocating individual GPs within a practice would invite a risk of spill over [22].

A major strength of this study is the well-defined study population and the large number of patients. The response rate for GP questionnaires was high. The data obtained in the registries were complete, as were data on GP participation in the CME.

The high response rate of 81.0 % minimises the risk of selection bias, as seen also by the similarity of the lung cancer patients in the control and the intervention group. However, patients who were not included due to GP non-response may differ from patients of responding GPs in respect of diagnostic intervals.

A potential risk of information bias exists due to GP recall bias. However, the GPs were asked to answer the questionnaire based on their electronic records. We would not expect such recall bias to be unevenly distributed between the two randomised groups although GPs in the intervention group who participated in the CME might estimate the intervals even longer than the control GPs because they had recently received an up-date on lung cancer symptoms and their awareness of such symptoms in the daily practice would hence be heightened. This bias will underestimate a possible effect of the intervention on the two intervals. This could explain the non-significant difference in the primary care interval between the two groups.

Unfortunately, only about half of the invited GPs participated in the CME. The correction for non-compliance addresses this problem, but the analyses increase the uncertainty of the estimates and the study may hence be underpowered because the low participation rate of GPs was duly catered for in our sample size calculation. Still, the risk of experiencing a long diagnostic interval was 13 % higher in the control group than in the intervention group that also participated in the CME. This means that CME combined with direct access to CT may have expedited diagnosis, but a larger study is needed to fully evaluate the effect as we cannot disprove the null-hypothesis.

The study may be underpowered due to two additional elements. The LDCT part of the intervention would have the most impact in the diagnosis of patients who present with false negative X-rays. A false negative X-ray constitutes a major risk of delay of diagnosis and may occur in approximately 20 % of patients preceding diagnosis. When taking this into account the sample size may have been five times as large than the one we calculated. However, the CME aimed at increasing the GPs awareness of early lung cancer and encouraging the GPs to refer more patients to test which together would decrease time to diagnosis. Furthermore, hoping for a stage shift in diagnosed lung cancers was maybe a bit optimistic. When planning the study we calculated that more lung cancer patients would have been diagnosed by the LDCT. If so these patients would constitute a larger proportion of the lung cancers from the intervention GPs and thereby increasing the chance of a stage shift.

The intervention GPs in this study were offered a 1 h lung cancer up-date. Those who agreed to participate may have been more interested in lung cancer, and this group of GPs may already have performed better in diagnosing lung cancer, which would potentially underestimate the effect of training if it was generalised. We did find that the patients of intervention GPs participating in the CME had much shorter intervals than patients of non-participating GPs. This either implies that the intervention was a success or that the intervention GPs who received CME already performed better than the rest of the intervention group.

The intervention group referred statistically insignificantly more patients to the fast-track pathway but the PPV for lung cancer was identical in the two groups of GPs. This may indicate that CME has a positive effect by making the GPs refer more patients to diagnostics. The PPV was the same although an extra CT-scan was an option, which may suggest that intervention GPs were able to find more cancer patients in their practices, maybe because of a greater awareness of lung cancer signs and symptoms.

The present study utilised low-dose CT as the diagnostic tool. For lung cancer, CT has a high sensitivity, but a lower specificity. This implies that the method involves a risk of patient distress because of the relatively high number of false positive scans. Furthermore, a widespread concern is the risk of cancer secondary to radiation from the low-dose CTs and the subsequent imaging used to evaluate positive screens. A US study from 2013 addresses this problem in connection with low-dose CT screening studies [35]. Based on epidemiological data on radiation exposure and assuming annual low-dose CT from age 55 to age 74 (20 scans), they estimate a lifetime attributable risk of lung cancer mortality of 0.07 % for males and 0.14 for females. One single low-dose CT utilises not even half of the total annual radiation exposure from natural and man-made sources. In addition, the group of patients referred to a low-dose CT may have a higher risk of having lung cancer or other important diseases than other groups of patients, and the small radiation dose (potential inducing a cancer 20–30 years later) may contribute only very little to the other risks these patients are facing.

### Generalisability

This Danish single-setting randomised, controlled trial with complete inclusion of patients holds the opportunity to generalise the study results to other settings in which general practice serves as the first line of healthcare.

### Comparison with relevant literature

In the present study, the median primary care interval was 16 days which is longer than the similar Danish interval in 2010 (median 7 days, IQI: 0–30) [33]. Whether this means that the diagnosis of lung cancer is less expedite in 2012–2013 than in 2010 is unknown, but we suggest that it may rather be because of increased awareness of lung cancer symptoms and early diagnosis and therefore an earlier first symptom presentation date listed in the questionnaire.

The patients listed at CME participating GPs has shorter diagnostic intervals than patients listed at non-participating GPs. These findings are in line with results from a British study in 2012 [5]. The study showed that a simple information campaign could educate physicians (and the public) and thereby induce change in behavior and increase the chest X-ray referral rate. The positive results on CME in this study holds potential for CME as a tool for earlier cancer diagnosis and need evaluation in a larger study concerning cancer CME in primary care. Such study has been conducted in Denmark and we are awaiting the results [36].

In the present study, 15 lung cancers were diagnosed in 648 direct CT scans from primary care (2.3 %) (24). Because of the symptomatic presentation, more lung cancers are diagnosed than by screening. In the US screening trial, lung cancer was diagnosed in 0.7 % of screenings [2], in the Danish screening trial the incidence was 0.8 % [3]. This suggests that use of CT scan in symptomatic patients performs better than screening.

## Conclusions

This randomised, controlled study with direct access to low-dose CT and CME intervention had no statistically significant effect on time to diagnosis or stage at diagnosis in lung cancer patients. However, it could have an effect in a larger scale study with more statistical power. Also, we do not know how direct CT will perform in terms of patient/doctor satisfaction and in connection with diagnosis of other lung diseases; these issues were beyond the scope of the present study. Direct access to low-dose CT scan may be an alternative to lung cancer screening. Furthermore, a recommendation of low-dose CT screening should consider offering symptomatic, unscreened patients access to CT directly from primary care. The positive results from the CME on early lung cancer diagnosis holds potential for further studies in this important field.

## Appendix 1

**Table 5:** Characteristics of the lung cancer patients for whom intervention and control GPs returned the questionnaire

	All patients		Intervention + questionnaire		Control + questionnaire		*P*-value
	n	(%)	n	(%)	n	(%)	
All: n	331		137	(80.1)	131	(81.9)	
Gender:							
Male	157	(47.4)	72	(52.6)	55	(42.0)	0.083^a^
Female	174	(52.6)	65	(47.4)	76	(58.0)	
Age:							
Mean (95 % CI)	69.4	(68.4–70.5)	69.5	(67.9–71.1)	69.2	(67.5–70.9)	0.614^b^
Range		40–97 yrs.		45–90 yrs.		44–86 yrs.	
40–59 yrs	61	(18.4)	26	(19.0)	23	(17.6)	0.821^c^
60–79 yrs	222	(67.0)	90	(65.7)	92	(70.2)	
80+	48	(14.5)	21	(15.3)	16	(12.2)	
Education:							
< 10 yrs	136	(41.1)	54	(39.4)	60	(45.8)	0.389^c^
10–15	146	(44.1)	61	(44.5)	54	(41.2)	
> 15 yrs	40	(12.1)	17	(12.4)	15	(11.5)	
Unknown	9	(2.7)	5	(3.7)	2	(1.5)	
Marital status							
Cohabitating	172	(52.0)	70	(51.1)	71	(54.2)	0.612^a^
Living alone	159	(48.0)	67	(48.9)	60	(45.8)	
Comorbidity^d^							
Yes	231	(69.8)	117	(85.4)	114	(87.0)	0.932^a^
No	35	(10.6)	18	(13.1)	17	(13.0)	
Unknown	65	(19.6)	2	(1.5)	0		

^a^Differences between groups were tested by Pearsons χ^2^ test. ^b^Age differences between groups were tested by Student’s T-test. ^c^Differences between groups were tested by Wilcoxon test. ^d^Data from GP questionnaire

## References

[1] Jemal A, Bray F, Center MM, Ferlay J, Ward E, Forman D (2011). Global cancer statistics. CA Cancer J Clin.

[2] Midthun DE, Jett JR. Screening for lung cancer: The US studies. J Surg Oncol. 2013;108(5):275-9. doi: 10.1002/jso.23391. Epub 2013 Aug 5. 10.1002/jso.23391 23918530

[3] Pedersen JH, Ashraf H, Dirksen A, Bach K, Hansen H, Toennesen P (2009). The Danish randomized lung cancer CT screening trial--overall design and results of the prevalence round. J Thorac Oncol.

[4] Campbell J, Pyer M, Rogers S, Walter D, Reddy R (2013). Enabling patients with respiratory symptoms to access chest X-rays on demand: the experience of the walk-in service in Corby, UK. J Public Health.

[5] Athey VL, Suckling RJ, Tod AM, Walters SJ, Rogers TK (2012). Early diagnosis of lung cancer: evaluation of a community-based social marketing intervention. Thorax.

[6] Smith S, Fielding S, Murchie P, Johnston M, Wyke S, Powell R (2013). Reducing the time before consulting with symptoms of lung cancer: a randomised controlled trial in primary care. Br J Gen Pract.

[7] Allgar VL, Neal RD (2005). Delays in the diagnosis of six cancers: analysis of data from the National Survey of NHS Patients: Cancer. Br J Cancer.

[8] Nielsen TN, Hansen RP, Vedsted P (2010). Symptom presentation in cancer patients in general practice. Ugeskr Laeger.

[9] Moth G, Olesen F, Vedsted P (2012). Reasons for encounter and disease patterns in Danish primary care - changes over 16 years. Scand J Prim Health Care.

[10] Hamilton W (2009). Five misconceptions in cancer diagnosis. Br J Gen Pract.

[11] Probst HB, Hussain ZB, Andersen O (2012). Cancer patient pathways in Denmark as a joint effort between bureaucrats, health professionals and politicians-A national Danish project. Health Policy.

[12] Olesen F, Hansen RP, Vedsted P (2009). Delay in diagnosis: the experience in Denmark. Br J Cancer.

[13] Hamilton W (2009). The CAPER studies: five case–control studies aimed at identifying and quantifying the risk of cancer in symptomatic primary care patients. Br J Cancer.

[14] Barrett J, Hamilton W (2008). Pathways to the diagnosis of lung cancer in the UK: a cohort study. BMC Fam Pract.

[15] Neal RD, Allgar VL, Ali N, Leese B, Heywood P, Proctor G (2007). Stage, survival and delays in lung, colorectal, prostate and ovarian cancer: comparison between diagnostic routes. Br J Gen Pract.

[16] Bjerager M (2006). Delay in diagnosis and treatment of lung cancer. PhD Thesis.

[17] Schneider J (2010). Early detection of lung cancers - Comparison of computed tomography, cytology and fuzzy-based tumor markers panels. Cancer Biomark.

[18] [www.lungecancer.dk]

[19] Hamilton W, Peters TJ, Round A, Sharp D (2005). What are the clinical features of lung cancer before the diagnosis is made? A population based case–control study. Thorax.

[20] MacMahon H, Austin JH, Gamsu G, Herold CJ, Jett JR, Naidich DP (2005). Fleischner Society: Guidelines for management of small pulmonary nodules detected on CT scans: a statement from the Fleischner Society. Radiology.

[21] Goldstraw P, Crowley J, Chansky K, Giroux DJ, Groome PA, Rami-Porta R (2007). International association for the study of lung cancer international staging committee, participating institutions: the IASLC lung cancer staging project: proposals for the revision of the TNM stage groupings in the forthcoming (seventh) edition of the TNM classification of malignant tumours. J Thorac Oncol.

[22] Campbell MK, Elbourne DR, Altman DG (2004). CONSORT statement: extension to cluster randomised trials. BMJ.

[23] Hansen RP, Vedsted P, Sokolowski I, Sondergaard J, Olesen F (2011). Time intervals from first symptom to treatment of cancer: a cohort study of 2,212 newly diagnosed cancer patients. BMC Health Serv Res.

[24] Guldbrandt LM, Rasmussen TR, Rasmussen F, Vedsted P (2014). Implementing direct access to Low-dose computed tomography in general practice-method adaption and outcome. PLoS One.

[25] Beyer M, Neal RD, Hiom S, Muth C, Nafees S, Van RE (2012). The Aarhus statement: improving design and reporting of studies on early cancer diagnosis. Br J Cancer.

[26] Pepek JM, Chino JP, Marks LB, D’amico TA, Yoo DS, Onaitis MW (2011). How well does the new lung cancer staging system predict for local/regional recurrence after surgery?: A comparison of the TNM 6 and 7 systems. J Thorac Oncol.

[27] Jakobsen E, Palshof T, Osterlind K, Pilegaard H (2009). Data from a national lung cancer registry contributes to improve outcome and quality of surgery: Danish results. Eur J Cardiothorac Surg.

[28] Lynge E, Sandegaard JL, Rebolj M (2011). The Danish national patient register. Scand J Public Health.

[29] [www.sst.dk]

[30] Timmermans B: The Danish Integrated Database for Labor Market Research: Towards Demystification for the English Speaking Audience. 2010, :1–17

[31] Pedersen CB (2011). The Danish civil registration system. Scand J Public Health.

[32] Larsen MB, Jensen H, Hansen RP, Olesen F, Vedsted P (2014). Identification of patients with incident cancers using administrative registry data. Dan Med J.

[33] Guldbrandt LM, Fenger-Grøn M, Rasmussen TR, Jensen H, Vedsted P (2015). The role of general practice in routes to diagnosis of lung cancer in Denmark: a population-based study of general practice involvement, diagnostic activity and diagnostic intervals. BMC Health Serv Res.

[34] Cuzick J, Edwards R, Segnan N (1997). Adjusting for non-compliance and contamination in randomized clinical trials. Stat Med.

[35] Frank L, Christodoulou E, Kazerooni EA (2013). Radiation risk of lung cancer screening. Semin Respir Crit Care Med.

[36] Toftegaard B, Bro F, Vedsted P (2014). A geographical cluster randomised stepped wedge study of continuing medical education and cancer diagnosis in general practice. Implement Sci.

